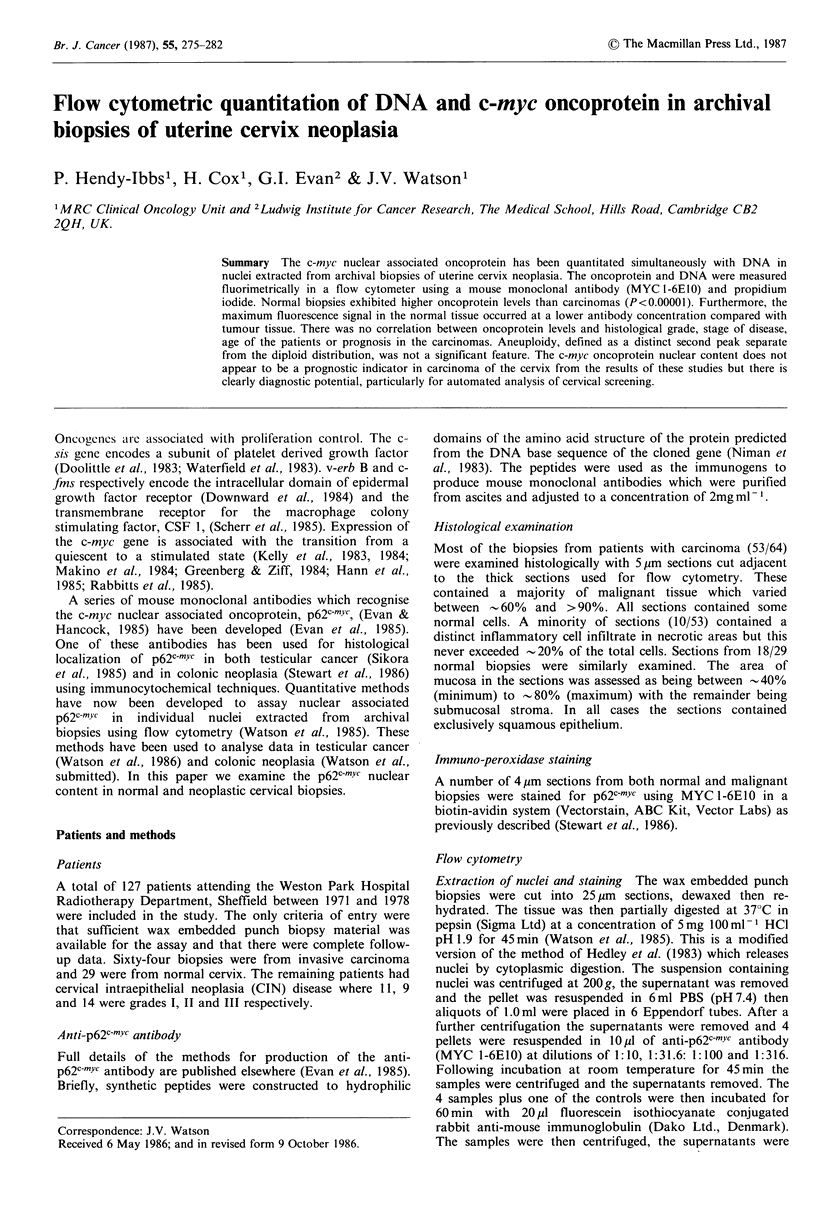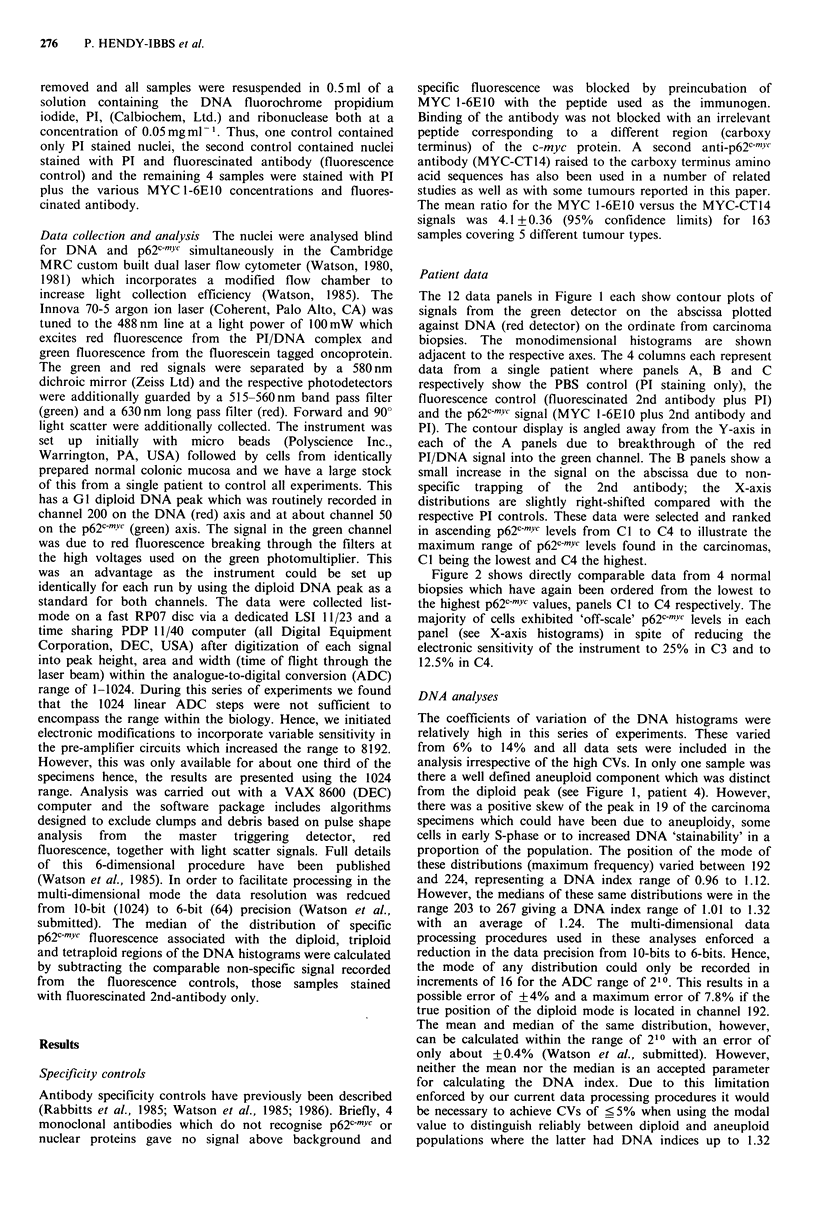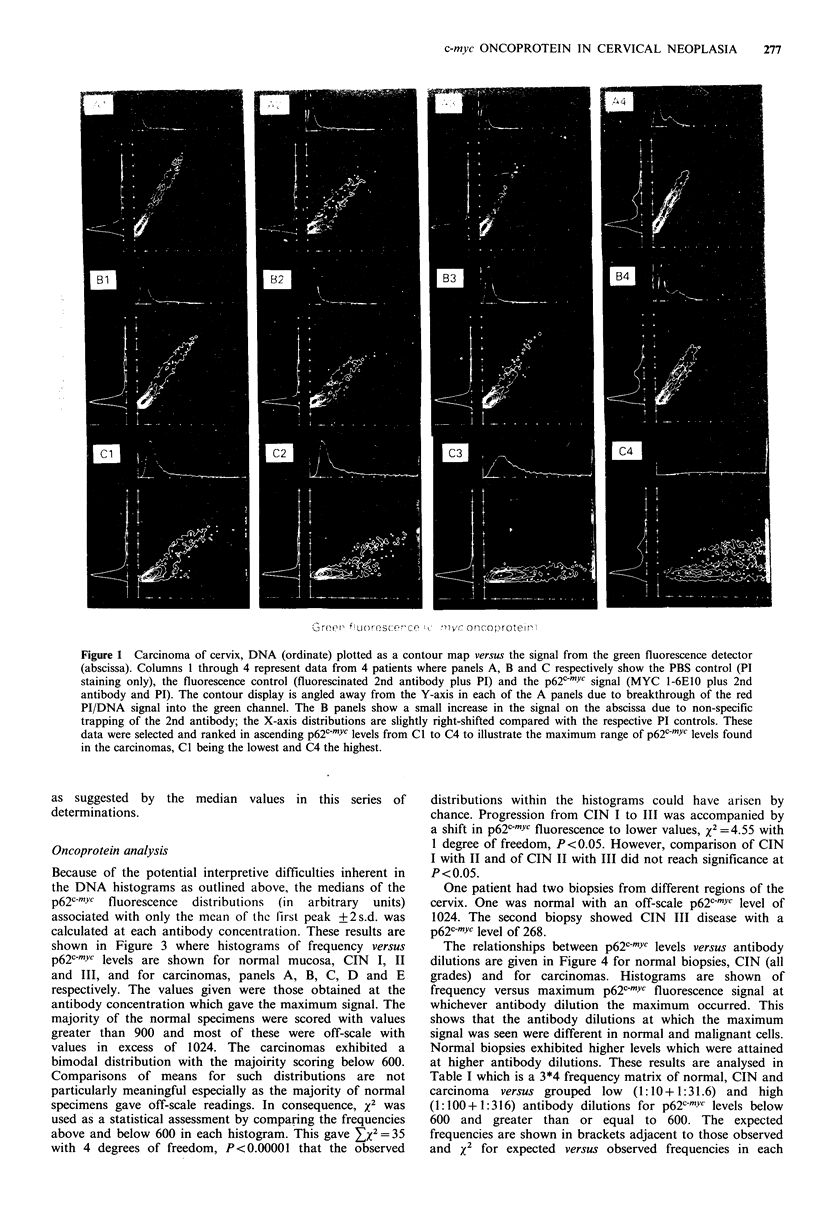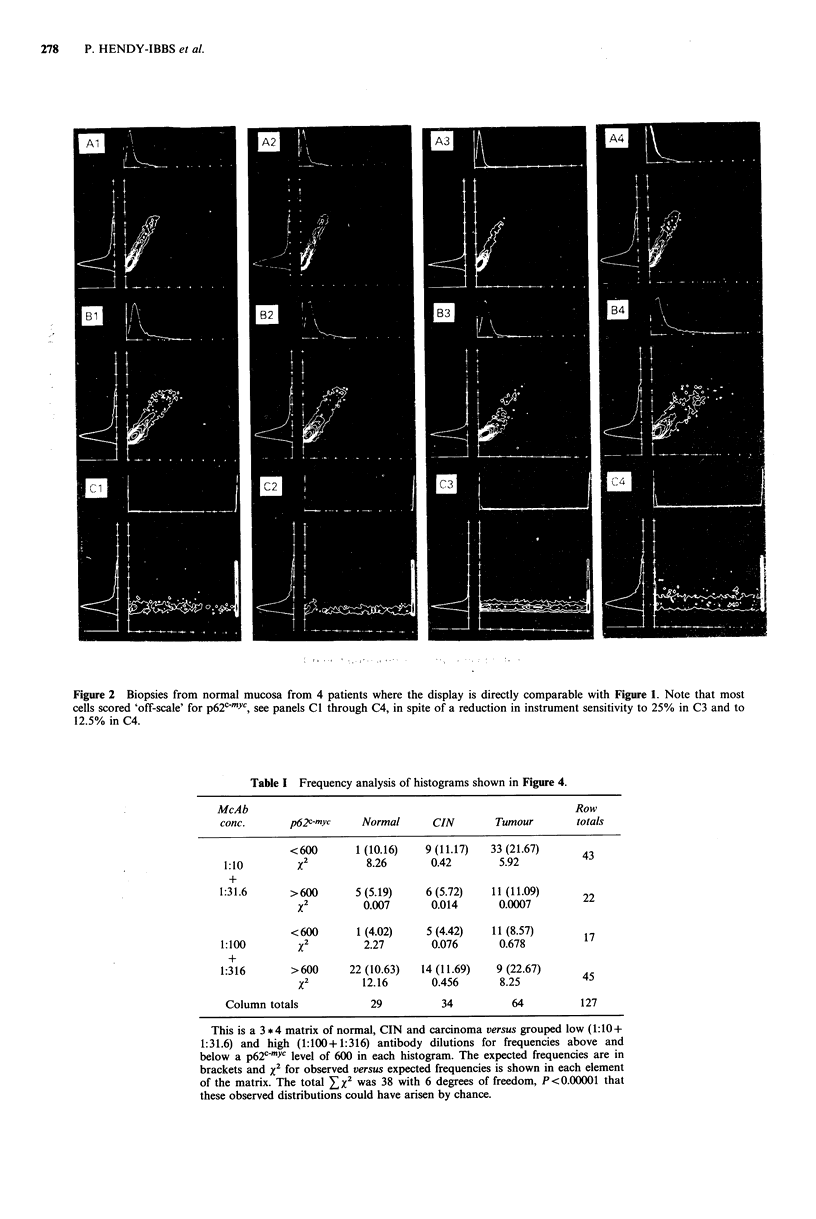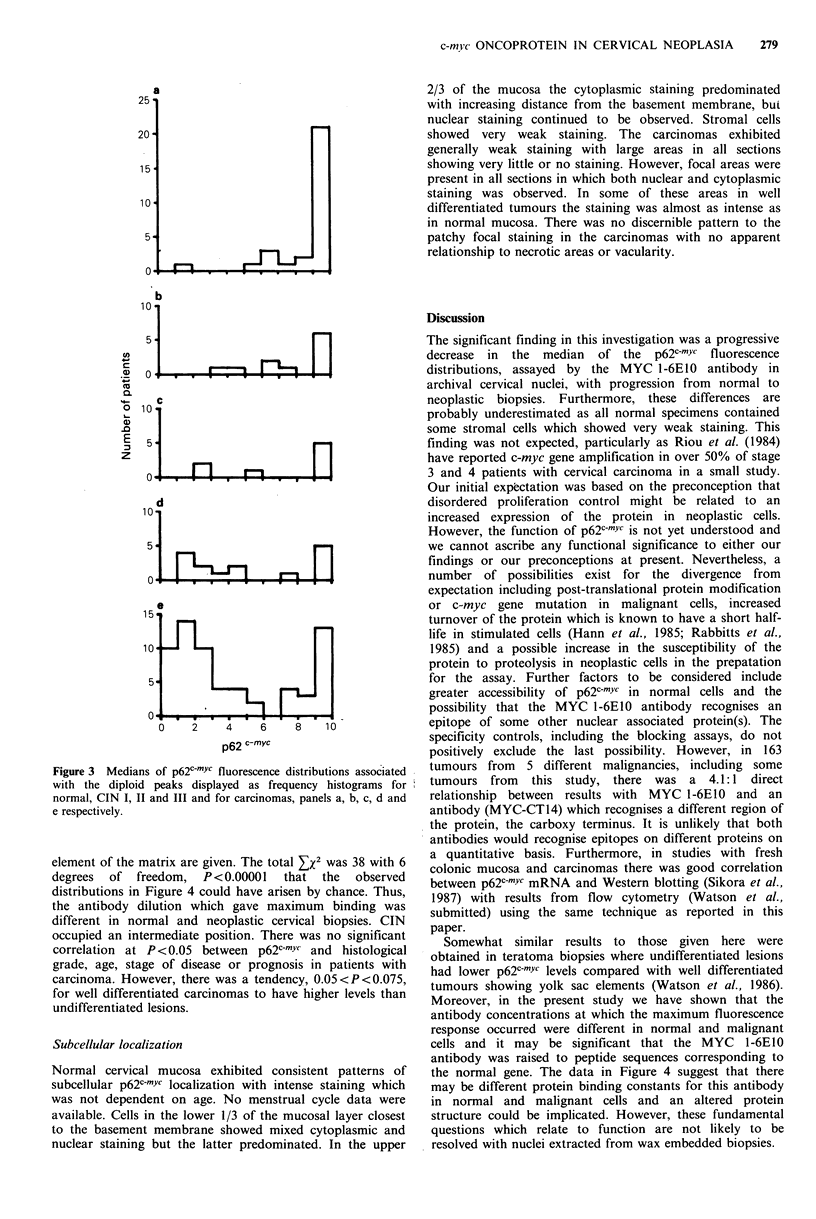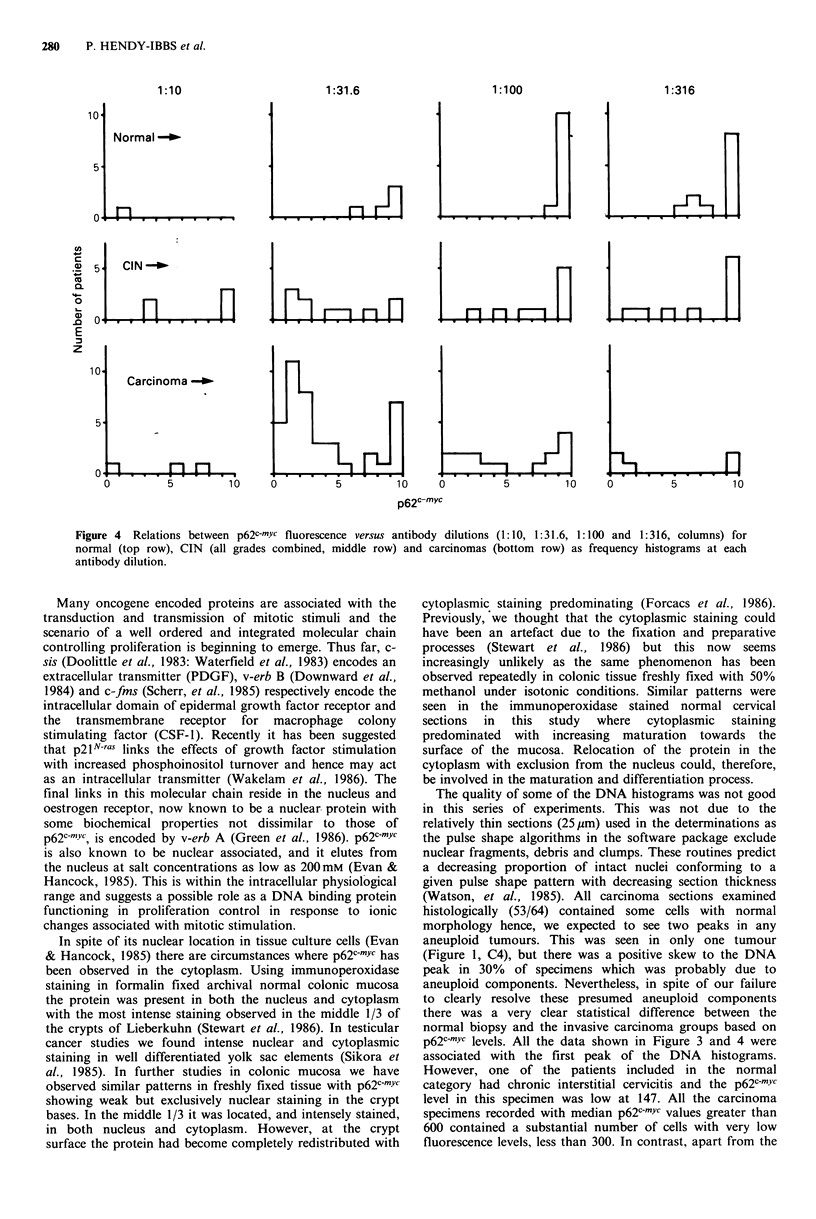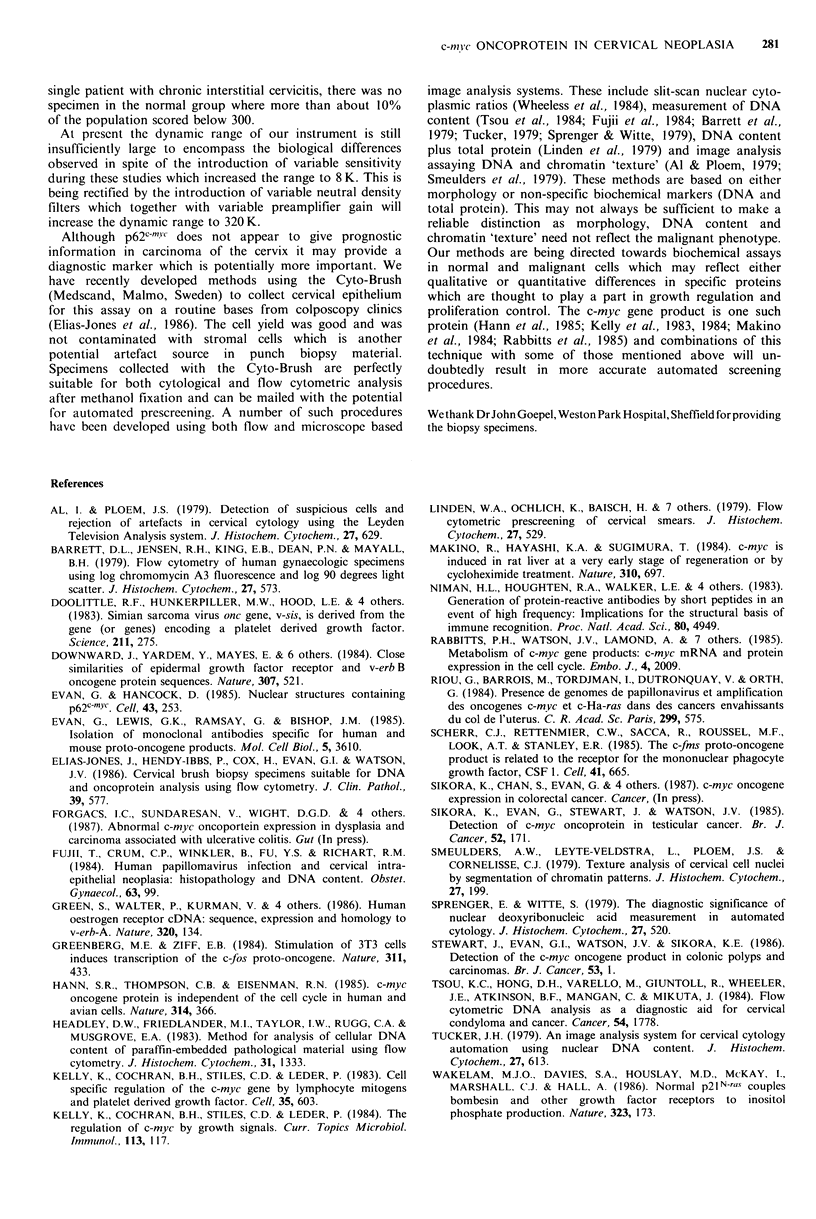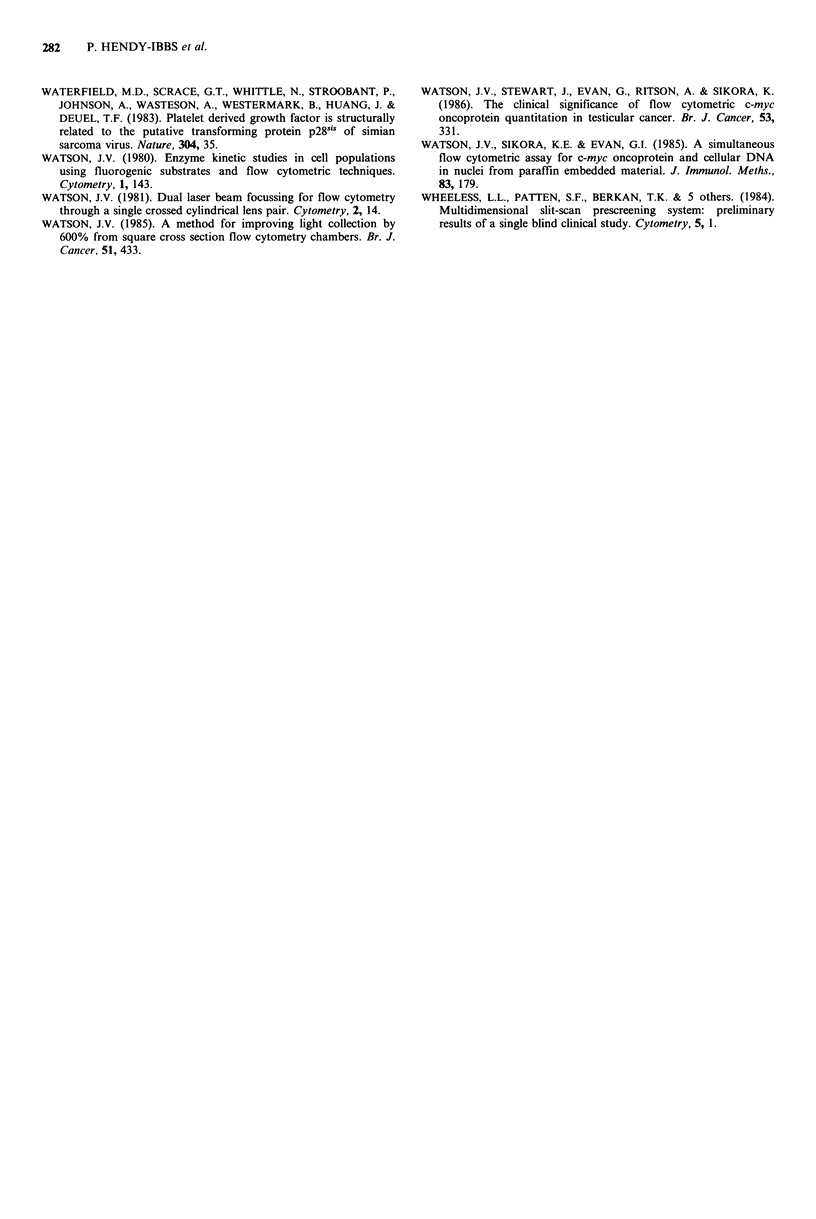# Flow cytometric quantitation of DNA and c-myc oncoprotein in archival biopsies of uterine cervix neoplasia.

**DOI:** 10.1038/bjc.1987.53

**Published:** 1987-03

**Authors:** P. Hendy-Ibbs, H. Cox, G. I. Evan, J. V. Watson

## Abstract

The c-myc nuclear associated oncoprotein has been quantitated simultaneously with DNA in nuclei extracted from archival biopsies of uterine cervix neoplasia. The oncoprotein and DNA were measured fluorimetrically in a flow cytometer using a mouse monoclonal antibody (MYC 1-6E10) and propidium iodide. Normal biopsies exhibited higher oncoprotein levels than carcinomas (P less than 0.00001). Furthermore, the maximum fluorescence signal in the normal tissue occurred at a lower antibody concentration compared with tumour tissue. There was no correlation between oncoprotein levels and histological grade, stage of disease, age of the patients or prognosis in the carcinomas. Aneuploidy, defined as a distinct second peak separate from the diploid distribution, was not a significant feature. The c-myc oncoprotein nuclear content does not appear to be a prognostic indicator in carcinoma of the cervix from the results of these studies but there is clearly diagnostic potential, particularly for automated analysis of cervical screening.


*Br. J. Cancer* (1987), **55**, 275–282 © The Macmillan Press Ltd., 1987

### Flow cytometric quantitation of DNA and c-*myc* oncoprotein in archival biopsies of uterine cervix neoplasia

P. Hendy-Ibbs[1], H. Cox[1], G.I. Evan[2] & J.V. Watson[1]

[1]*MRC Clinical Oncology Unit and* [2]*Ludwig Institute for Cancer Research, The Medical School, Hills Road, Cambridge CB2 2QH, UK.*

**Summary** The c-*myc* nuclear associated oncoprotein has been quantitated simultaneously with DNA in nuclei extracted from archival biopsies of uterine cervix neoplasia. The oncoprotein and DNA were measured fluorimetrically in a flow cytometer using a mouse monoclonal antibody (MYC 1-6E10) and propidium iodide. Normal biopsies exhibited higher oncoprotein levels than carcinomas ($P<0.00001$). Furthermore, the maximum fluorescence signal in the normal tissue occurred at a lower antibody concentration compared with tumour tissue. There was no correlation between oncoprotein levels and histological grade, stage of disease, age of the patients or prognosis in the carcinomas. Aneuploidy, defined as a distinct second peak separate from the diploid distribution, was not a significant feature. The c-*myc* oncoprotein nuclear content does not appear to be a prognostic indicator in carcinoma of the cervix from the results of these studies but there is clearly diagnostic potential, particularly for automated analysis of cervical screening.

Oncogenes are associated with proliferation control. The c-*sis* gene encodes a subunit of platelet derived growth factor (Doolittle *et al.*, 1983; Waterfield *et al.*, 1983). v-*erb* B and c-*fms* respectively encode the intracellular domain of epidermal growth factor receptor (Downward *et al.*, 1984) and the transmembrane receptor for the macrophage colony stimulating factor, CSF 1, (Scherr *et al.*, 1985). Expression of the c-*myc* gene is associated with the transition from a quiescent to a stimulated state (Kelly *et al.*, 1983, 1984; Makino *et al.*, 1984; Greenberg & Ziff, 1984; Hann *et al.*, 1985; Rabbitts *et al.*, 1985).

A series of mouse monoclonal antibodies which recognise the c-*myc* nuclear associated oncoprotein, $p62^{c\text{-}myc}$, (Evan & Hancock, 1985) have been developed (Evan *et al.*, 1985). One of these antibodies has been used for histological localization of $p62^{c\text{-}myc}$ in both testicular cancer (Sikora *et al.*, 1985) and in colonic neoplasia (Stewart *et al.*, 1986) using immunocytochemical techniques. Quantitative methods have now been developed to assay nuclear associated $p62^{c\text{-}myc}$ in individual nuclei extracted from archival biopsies using flow cytometry (Watson *et al.*, 1985). These methods have been used to analyse data in testicular cancer (Watson *et al.*, 1986) and colonic neoplasia (Watson *et al.*, submitted). In this paper we examine the $p62^{c\text{-}myc}$ nuclear content in normal and neoplastic cervical biopsies.

### Patients and methods

### *Patients*

A total of 127 patients attending the Weston Park Hospital Radiotherapy Department, Sheffield between 1971 and 1978 were included in the study. The only criteria of entry were that sufficient wax embedded punch biopsy material was available for the assay and that there were complete follow-up data. Sixty-four biopsies were from invasive carcinoma and 29 were from normal cervix. The remaining patients had cervical intraepithelial neoplasia (CIN) disease where 11, 9 and 14 were grades I, II and III respectively.

### *Anti-*$p62^{c\text{-}myc}$ *antibody*

Full details of the methods for production of the anti-$p62^{c\text{-}myc}$ antibody are published elsewhere (Evan *et al.*, 1985). Briefly, synthetic peptides were constructed to hydrophilic domains of the amino acid structure of the protein predicted from the DNA base sequence of the cloned gene (Niman *et al.*, 1983). The peptides were used as the immunogens to produce mouse monoclonal antibodies which were purified from ascites and adjusted to a concentration of $2 mg ml^{-1}$.

### *Histological examination*

Most of the biopsies from patients with carcinoma (53/64) were examined histologically with $5 \mu m$ sections cut adjacent to the thick sections used for flow cytometry. These contained a majority of malignant tissue which varied between $\sim 60\%$ and $>90\%$. All sections contained some normal cells. A minority of sections (10/53) contained a distinct inflammatory cell infiltrate in necrotic areas but this never exceeded $\sim 20\%$ of the total cells. Sections from 18/29 normal biopsies were similarly examined. The area of mucosa in the sections was assessed as being between $\sim 40\%$ (minimum) to $\sim 80\%$ (maximum) with the remainder being submucosal stroma. In all cases the sections contained exclusively squamous epithelium.

### *Immuno-peroxidase staining*

A number of $4 \mu m$ sections from both normal and malignant biopsies were stained for $p62^{c\text{-}myc}$ using MYC 1-6E10 in a biotin-avidin system (Vectorstain, ABC Kit, Vector Labs) as previously described (Stewart *et al.*, 1986).

### *Flow cytometry*

*Extraction of nuclei and staining* The wax embedded punch biopsies were cut into $25 \mu m$ sections, dewaxed then re-hydrated. The tissue was then partially digested at 37°C in pepsin (Sigma Ltd) at a concentration of $5 mg\ 100 ml^{-1}$ HCl pH 1.9 for 45 min (Watson *et al.*, 1985). This is a modified version of the method of Hedley *et al.* (1983) which releases nuclei by cytoplasmic digestion. The suspension containing nuclei was centrifuged at 200 *g*, the supernatant was removed and the pellet was resuspended in 6 ml PBS (pH 7.4) then aliquots of 1.0 ml were placed in 6 Eppendorf tubes. After a further centrifugation the supernatants were removed and 4 pellets were resuspended in $10 \mu l$ of anti-$p62^{c\text{-}myc}$ antibody (MYC 1-6E10) at dilutions of 1:10, 1:31.6: 1:100 and 1:316. Following incubation at room temperature for 45 min the samples were centrifuged and the supernatants removed. The 4 samples plus one of the controls were then incubated for 60 min with $20 \mu l$ fluorescein isothiocyanate conjugated rabbit anti-mouse immunoglobulin (Dako Ltd., Denmark). The samples were then centrifuged, the supernatants were

Correspondence: J.V. Watson
Received 6 May 1986; and in revised form 9 October 1986.

removed and all samples were resuspended in 0.5 ml of a solution containing the DNA fluorochrome propidium iodide, PI, (Calbiochem, Ltd.) and ribonuclease both at a concentration of $0.05 \, mg \, ml^{-1}$. Thus, one control contained only PI stained nuclei, the second control contained nuclei stained with PI and fluorescinated antibody (fluorescence control) and the remaining 4 samples were stained with PI plus the various MYC 1-6E10 concentrations and fluorescinated antibody.

*Data collection and analysis* The nuclei were analysed blind for DNA and $p62^{c\text{-}myc}$ simultaneously in the Cambridge MRC custom built dual laser flow cytometer (Watson, 1980, 1981) which incorporates a modified flow chamber to increase light collection efficiency (Watson, 1985). The Innova 70-5 argon ion laser (Coherent, Palo Alto, CA) was tuned to the 488 nm line at a light power of 100 mW which excites red fluorescence from the PI/DNA complex and green fluorescence from the fluorescein tagged oncoprotein. The green and red signals were separated by a 580 nm dichroic mirror (Zeiss Ltd) and the respective photodetectors were additionally guarded by a 515–560 nm band pass filter (green) and a 630 nm long pass filter (red). Forward and 90° light scatter were additionally collected. The instrument was set up initially with micro beads (Polyscience Inc., Warrington, PA, USA) followed by cells from identically prepared normal colonic mucosa and we have a large stock of this from a single patient to control all experiments. This has a G1 diploid DNA peak which was routinely recorded in channel 200 on the DNA (red) axis and at about channel 50 on the $p62^{c\text{-}myc}$ (green) axis. The signal in the green channel was due to red fluorescence breaking through the filters at the high voltages used on the green photomultiplier. This was an advantage as the instrument could be set up identically for each run by using the diploid DNA peak as a standard for both channels. The data were collected list-mode on a fast RP07 disc via a dedicated LSI 11/23 and a time sharing PDP 11/40 computer (all Digital Equipment Corporation, DEC, USA) after digitization of each signal into peak height, area and width (time of flight through the laser beam) within the analogue-to-digital conversion (ADC) range of 1–1024. During this series of experiments we found that the 1024 linear ADC steps were not sufficient to encompass the range within the biology. Hence, we initiated electronic modifications to incorporate variable sensitivity in the pre-amplifier circuits which increased the range to 8192. However, this was only available for about one third of the specimens hence, the results are presented using the 1024 range. Analysis was carried out with a VAX 8600 (DEC) computer and the software package includes algorithms designed to exclude clumps and debris based on pulse shape analysis from the master triggering detector, red fluorescence, together with light scatter signals. Full details of this 6-dimensional procedure have been published (Watson *et al.*, 1985). In order to facilitate processing in the multi-dimensional mode the data resolution was redcued from 10-bit (1024) to 6-bit (64) precision (Watson *et al.*, submitted). The median of the distribution of specific $p62^{c\text{-}myc}$ fluorescence associated with the diploid, triploid and tetraploid regions of the DNA histograms were calculated by subtracting the comparable non-specific signal recorded from the fluorescence controls, those samples stained with fluorescinated 2nd-antibody only.

### Results

*Specificity controls*

Antibody specificity controls have previously been described (Rabbitts *et al.*, 1985; Watson *et al.*, 1985; 1986). Briefly, 4 monoclonal antibodies which do not recognise $p62^{c\text{-}myc}$ or nuclear proteins gave no signal above background and specific fluorescence was blocked by preincubation of MYC 1-6E10 with the peptide used as the immunogen. Binding of the antibody was not blocked with an irrelevant peptide corresponding to a different region (carboxy terminus) of the *c-myc* protein. A second anti-$p62^{c\text{-}myc}$ antibody (MYC-CT14) raised to the carboxy terminus amino acid sequences has also been used in a number of related studies as well as with some tumours reported in this paper. The mean ratio for the MYC 1-6E10 versus the MYC-CT14 signals was $4.1 \pm 0.36$ (95% confidence limits) for 163 samples covering 5 different tumour types.

*Patient data*

The 12 data panels in Figure 1 each show contour plots of signals from the green detector on the abscissa plotted against DNA (red detector) on the ordinate from carcinoma biopsies. The monodimensional histograms are shown adjacent to the respective axes. The 4 columns each represent data from a single patient where panels A, B and C respectively show the PBS control (PI staining only), the fluorescence control (fluorescinated 2nd antibody plus PI) and the $p62^{c\text{-}myc}$ signal (MYC 1-6E10 plus 2nd antibody and PI). The contour display is angled away from the Y-axis in each of the A panels due to breakthrough of the red PI/DNA signal into the green channel. The B panels show a small increase in the signal on the abscissa due to non-specific trapping of the 2nd antibody; the X-axis distributions are slightly right-shifted compared with the respective PI controls. These data were selected and ranked in ascending $p62^{c\text{-}myc}$ levels from C1 to C4 to illustrate the maximum range of $p62^{c\text{-}myc}$ levels found in the carcinomas, C1 being the lowest and C4 the highest.

Figure 2 shows directly comparable data from 4 normal biopsies which have again been ordered from the lowest to the highest $p62^{c\text{-}myc}$ values, panels C1 to C4 respectively. The majority of cells exhibited 'off-scale' $p62^{c\text{-}myc}$ levels in each panel (see X-axis histograms) in spite of reducing the electronic sensitivity of the instrument to 25% in C3 and to 12.5% in C4.

*DNA analyses*

The coefficients of variation of the DNA histograms were relatively high in this series of experiments. These varied from 6% to 14% and all data sets were included in the analysis irrespective of the high CVs. In only one sample was there a well defined aneuploid component which was distinct from the diploid peak (see Figure 1, patient 4). However, there was a positive skew of the peak in 19 of the carcinoma specimens which could have been due to aneuploidy, some cells in early S-phase or to increased DNA 'stainability' in a proportion of the population. The position of the mode of these distributions (maximum frequency) varied between 192 and 224, representing a DNA index range of 0.96 to 1.12. However, the medians of these same distributions were in the range 203 to 267 giving a DNA index range of 1.01 to 1.32 with an average of 1.24. The multi-dimensional data processing procedures used in these analyses enforced a reduction in the data precision from 10-bits to 6-bits. Hence, the mode of any distribution could only be recorded in increments of 16 for the ADC range of $2^{10}$. This results in a possible error of $\pm 4\%$ and a maximum error of 7.8% if the true position of the diploid mode is located in channel 192. The mean and median of the same distribution, however, can be calculated within the range of $2^{10}$ with an error of only about $\pm 0.4\%$ (Watson *et al.*, submitted). However, neither the mean nor the median is an accepted parameter for calculating the DNA index. Due to this limitation enforced by our current data processing procedures it would be necessary to achieve CVs of $\leq 5\%$ when using the modal value to distinguish reliably between diploid and aneuploid populations where the latter had DNA indices up to 1.32

**Figure 1** Carcinoma of cervix, DNA (ordinate) plotted as a contour map *versus* the signal from the green fluorescence detector (abscissa). Columns 1 through 4 represent data from 4 patients where panels A, B and C respectively show the PBS control (PI staining only), the fluorescence control (fluorescinated 2nd antibody plus PI) and the $p62^{c\text{-}myc}$ signal (MYC 1-6E10 plus 2nd antibody and PI). The contour display is angled away from the Y-axis in each of the A panels due to breakthrough of the red PI/DNA signal into the green channel. The B panels show a small increase in the signal on the abscissa due to non-specific trapping of the 2nd antibody; the X-axis distributions are slightly right-shifted compared with the respective PI controls. These data were selected and ranked in ascending $p62^{c\text{-}myc}$ levels from C1 to C4 to illustrate the maximum range of $p62^{c\text{-}myc}$ levels found in the carcinomas, C1 being the lowest and C4 the highest.

as suggested by the median values in this series of determinations.

*Oncoprotein analysis*

Because of the potential interpretive difficulties inherent in the DNA histograms as outlined above, the medians of the $p62^{c\text{-}myc}$ fluorescence distributions (in arbitrary units) associated with only the mean of the first peak $\pm 2$ s.d. was calculated at each antibody concentration. These results are shown in Figure 3 where histograms of frequency *versus* $p62^{c\text{-}myc}$ levels are shown for normal mucosa, CIN I, II and III, and for carcinomas, panels A, B, C, D and E respectively. The values given were those obtained at the antibody concentration which gave the maximum signal. The majority of the normal specimens were scored with values greater than 900 and most of these were off-scale with values in excess of 1024. The carcinomas exhibited a bimodal distribution with the majoirity scoring below 600. Comparisons of means for such distributions are not particularly meaningful especially as the majority of normal specimens gave off-scale readings. In consequence, $\chi^2$ was used as a statistical assessment by comparing the frequencies above and below 600 in each histogram. This gave $\sum\chi^2 = 35$ with 4 degrees of freedom, $P < 0.00001$ that the observed distributions within the histograms could have arisen by chance. Progression from CIN I to III was accompanied by a shift in $p62^{c\text{-}myc}$ fluorescence to lower values, $\chi^2 = 4.55$ with 1 degree of freedom, $P < 0.05$. However, comparison of CIN I with II and of CIN II with III did not reach significance at $P < 0.05$.

One patient had two biopsies from different regions of the cervix. One was normal with an off-scale $p62^{c\text{-}myc}$ level of 1024. The second biopsy showed CIN III disease with a $p62^{c\text{-}myc}$ level of 268.

The relationships between $p62^{c\text{-}myc}$ levels *versus* antibody dilutions are given in Figure 4 for normal biopsies, CIN (all grades) and for carcinomas. Histograms are shown of frequency versus maximum $p62^{c\text{-}myc}$ fluorescence signal at whichever antibody dilution the maximum occurred. This shows that the antibody dilutions at which the maximum signal was seen were different in normal and malignant cells. Normal biopsies exhibited higher levels which were attained at higher antibody dilutions. These results are analysed in Table I which is a 3*4 frequency matrix of normal, CIN and carcinoma *versus* grouped low (1:10+1:31.6) and high (1:100+1:316) antibody dilutions for $p62^{c\text{-}myc}$ levels below 600 and greater than or equal to 600. The expected frequencies are shown in brackets adjacent to those observed and $\chi^2$ for expected *versus* observed frequencies in each

**Figure 2** Biopsies from normal mucosa from 4 patients where the display is directly comparable with **Figure 1**. Note that most cells scored 'off-scale' for $p62^{c\text{-}myc}$, see panels C1 through C4, in spite of a reduction in instrument sensitivity to 25% in C3 and to 12.5% in C4.

**Table I** Frequency analysis of histograms shown in **Figure 4**.

| *McAb conc.* | $p62^{c\text{-}myc}$ | *Normal* | *CIN* | *Tumour* | *Row totals* |
|---|---|---|---|---|---|
| 1:10 + 1:31.6 | <600 | 1 (10.16) | 9 (11.17) | 33 (21.67) | 43 |
| | $\chi^2$ | 8.26 | 0.42 | 5.92 | |
| | >600 | 5 (5.19) | 6 (5.72) | 11 (11.09) | 22 |
| | $\chi^2$ | 0.007 | 0.014 | 0.0007 | |
| 1:100 + 1:316 | <600 | 1 (4.02) | 5 (4.42) | 11 (8.57) | 17 |
| | $\chi^2$ | 2.27 | 0.076 | 0.678 | |
| | >600 | 22 (10.63) | 14 (11.69) | 9 (22.67) | 45 |
| | $\chi^2$ | 12.16 | 0.456 | 8.25 | |
| Column totals | | 29 | 34 | 64 | 127 |

This is a 3 * 4 matrix of normal, CIN and carcinoma *versus* grouped low (1:10+1:31.6) and high (1:100+1:316) antibody dilutions for frequencies above and below a $p62^{c\text{-}myc}$ level of 600 in each histogram. The expected frequencies are in brackets and $\chi^2$ for observed *versus* expected frequencies is shown in each element of the matrix. The total $\sum\chi^2$ was 38 with 6 degrees of freedom, $P<0.00001$ that these observed distributions could have arisen by chance.

**Figure 3** Medians of p62$^{c\text{-}myc}$ fluorescence distributions associated with the diploid peaks displayed as frequency histograms for normal, CIN I, II and III and for carcinomas, panels a, b, c, d and e respectively.

element of the matrix are given. The total $\sum\chi^2$ was 38 with 6 degrees of freedom, $P<0.00001$ that the observed distributions in Figure 4 could have arisen by chance. Thus, the antibody dilution which gave maximum binding was different in normal and neoplastic cervical biopsies. CIN occupied an intermediate position. There was no significant correlation at $P<0.05$ between p62$^{c\text{-}myc}$ and histological grade, age, stage of disease or prognosis in patients with carcinoma. However, there was a tendency, $0.05<P<0.075$, for well differentiated carcinomas to have higher levels than undifferentiated lesions.

### *Subcellular localization*

Normal cervical mucosa exhibited consistent patterns of subcellular p62$^{c\text{-}myc}$ localization with intense staining which was not dependent on age. No menstrual cycle data were available. Cells in the lower 1/3 of the mucosal layer closest to the basement membrane showed mixed cytoplasmic and nuclear staining but the latter predominated. In the upper 2/3 of the mucosa the cytoplasmic staining predominated with increasing distance from the basement membrane, but nuclear staining continued to be observed. Stromal cells showed very weak staining. The carcinomas exhibited generally weak staining with large areas in all sections showing very little or no staining. However, focal areas were present in all sections in which both nuclear and cytoplasmic staining was observed. In some of these areas in well differentiated tumours the staining was almost as intense as in normal mucosa. There was no discernible pattern to the patchy focal staining in the carcinomas with no apparent relationship to necrotic areas or vacularity.

### Discussion

The significant finding in this investigation was a progressive decrease in the median of the p62$^{c\text{-}myc}$ fluorescence distributions, assayed by the MYC 1-6E10 antibody in archival cervical nuclei, with progression from normal to neoplastic biopsies. Furthermore, these differences are probably underestimated as all normal specimens contained some stromal cells which showed very weak staining. This finding was not expected, particularly as Riou *et al.* (1984) have reported c-*myc* gene amplification in over 50% of stage 3 and 4 patients with cervical carcinoma in a small study. Our initial expectation was based on the preconception that disordered proliferation control might be related to an increased expression of the protein in neoplastic cells. However, the function of p62$^{c\text{-}myc}$ is not yet understood and we cannot ascribe any functional significance to either our findings or our preconceptions at present. Nevertheless, a number of possibilities exist for the divergence from expectation including post-translational protein modification or c-*myc* gene mutation in malignant cells, increased turnover of the protein which is known to have a short half-life in stimulated cells (Hann *et al.*, 1985; Rabbitts *et al.*, 1985) and a possible increase in the susceptibility of the protein to proteolysis in neoplastic cells in the prepatation for the assay. Further factors to be considered include greater accessibility of p62$^{c\text{-}myc}$ in normal cells and the possibility that the MYC 1-6E10 antibody recognises an epitope of some other nuclear associated protein(s). The specificity controls, including the blocking assays, do not positively exclude the last possibility. However, in 163 tumours from 5 different malignancies, including some tumours from this study, there was a 4.1:1 direct relationship between results with MYC 1-6E10 and an antibody (MYC-CT14) which recognises a different region of the protein, the carboxy terminus. It is unlikely that both antibodies would recognise epitopes on different proteins on a quantitative basis. Furthermore, in studies with fresh colonic mucosa and carcinomas there was good correlation between p62$^{c\text{-}myc}$ mRNA and Western blotting (Sikora *et al.*, 1987) with results from flow cytometry (Watson *et al.*, submitted) using the same technique as reported in this paper.

Somewhat similar results to those given here were obtained in teratoma biopsies where undifferentiated lesions had lower p62$^{c\text{-}myc}$ levels compared with well differentiated tumours showing yolk sac elements (Watson *et al.*, 1986). Moreover, in the present study we have shown that the antibody concentrations at which the maximum fluorescence response occurred were different in normal and malignant cells and it may be significant that the MYC 1-6E10 antibody was raised to peptide sequences corresponding to the normal gene. The data in Figure 4 suggest that there may be different protein binding constants for this antibody in normal and malignant cells and an altered protein structure could be implicated. However, these fundamental questions which relate to function are not likely to be resolved with nuclei extracted from wax embedded biopsies.

**Figure 4** Relations between p62$^{c\text{-}myc}$ fluorescence *versus* antibody dilutions (1:10, 1:31.6, 1:100 and 1:316, columns) for normal (top row), CIN (all grades combined, middle row) and carcinomas (bottom row) as frequency histograms at each antibody dilution.

Many oncogene encoded proteins are associated with the transduction and transmission of mitotic stimuli and the scenario of a well ordered and integrated molecular chain controlling proliferation is beginning to emerge. Thus far, c-*sis* (Doolittle *et al.*, 1983: Waterfield *et al.*, 1983) encodes an extracellular transmitter (PDGF), v-*erb* B (Downward *et al.*, 1984) and c-*fms* (Scherr, *et al.*, 1985) respectively encode the intracellular domain of epidermal growth factor receptor and the transmembrane receptor for macrophage colony stimulating factor (CSF-1). Recently it has been suggested that p21$^{N\text{-}ras}$ links the effects of growth factor stimulation with increased phosphoinositol turnover and hence may act as an intracellular transmitter (Wakelam *et al.*, 1986). The final links in this molecular chain reside in the nucleus and oestrogen receptor, now known to be a nuclear protein with some biochemical properties not dissimilar to those of p62$^{c\text{-}myc}$, is encoded by v-*erb* A (Green *et al.*, 1986). p62$^{c\text{-}myc}$ is also known to be nuclear associated, and it elutes from the nucleus at salt concentrations as low as 200 mM (Evan & Hancock, 1985). This is within the intracellular physiological range and suggests a possible role as a DNA binding protein functioning in proliferation control in response to ionic changes associated with mitotic stimulation.

In spite of its nuclear location in tissue culture cells (Evan & Hancock, 1985) there are circumstances where p62$^{c\text{-}myc}$ has been observed in the cytoplasm. Using immunoperoxidase staining in formalin fixed archival normal colonic mucosa the protein was present in both the nucleus and cytoplasm with the most intense staining observed in the middle 1/3 of the crypts of Lieberkuhn (Stewart *et al.*, 1986). In testicular cancer studies we found intense nuclear and cytoplasmic staining in well differentiated yolk sac elements (Sikora *et al.*, 1985). In further studies in colonic mucosa we have observed similar patterns in freshly fixed tissue with p62$^{c\text{-}myc}$ showing weak but exclusively nuclear staining in the crypt bases. In the middle 1/3 it was located, and intensely stained, in both nucleus and cytoplasm. However, at the crypt surface the protein had become completely redistributed with cytoplasmic staining predominating (Forcacs *et al.*, 1986). Previously, we thought that the cytoplasmic staining could have been an artefact due to the fixation and preparative processes (Stewart *et al.*, 1986) but this now seems increasingly unlikely as the same phenomenon has been observed repeatedly in colonic tissue freshly fixed with 50% methanol under isotonic conditions. Similar patterns were seen in the immunoperoxidase stained normal cervical sections in this study where cytoplasmic staining predominated with increasing maturation towards the surface of the mucosa. Relocation of the protein in the cytoplasm with exclusion from the nucleus could, therefore, be involved in the maturation and differentiation process.

The quality of some of the DNA histograms was not good in this series of experiments. This was not due to the relatively thin sections (25 $\mu$m) used in the determinations as the pulse shape algorithms in the software package exclude nuclear fragments, debris and clumps. These routines predict a decreasing proportion of intact nuclei conforming to a given pulse shape pattern with decreasing section thickness (Watson, *et al.*, 1985). All carcinoma sections examined histologically (53/64) contained some cells with normal morphology hence, we expected to see two peaks in any aneuploid tumours. This was seen in only one tumour (Figure 1, C4), but there was a positive skew to the DNA peak in 30% of specimens which was probably due to aneuploid components. Nevertheless, in spite of our failure to clearly resolve these presumed aneuploid components there was a very clear statistical difference between the normal biopsy and the invasive carcinoma groups based on p62$^{c\text{-}myc}$ levels. All the data shown in Figure 3 and 4 were associated with the first peak of the DNA histograms. However, one of the patients included in the normal category had chronic interstitial cervicitis and the p62$^{c\text{-}myc}$ level in this specimen was low at 147. All the carcinoma specimens recorded with median p62$^{c\text{-}myc}$ values greater than 600 contained a substantial number of cells with very low fluorescence levels, less than 300. In contrast, apart from the single patient with chronic interstitial cervicitis, there was no specimen in the normal group where more than about 10% of the population scored below 300.

At present the dynamic range of our instrument is still insufficiently large to encompass the biological differences observed in spite of the introduction of variable sensitivity during these studies which increased the range to 8 K. This is being rectified by the introduction of variable neutral density filters which together with variable preamplifier gain will increase the dynamic range to 320 K.

Although $p62^{c\text{-}myc}$ does not appear to give prognostic information in carcinoma of the cervix it may provide a diagnostic marker which is potentially more important. We have recently developed methods using the Cyto-Brush (Medscand, Malmo, Sweden) to collect cervical epithelium for this assay on a routine bases from colposcopy clinics (Elias-Jones *et al.*, 1986). The cell yield was good and was not contaminated with stromal cells which is another potential artefact source in punch biopsy material. Specimens collected with the Cyto-Brush are perfectly suitable for both cytological and flow cytometric analysis after methanol fixation and can be mailed with the potential for automated prescreening. A number of such procedures have been developed using both flow and microscope based image analysis systems. These include slit-scan nuclear cytoplasmic ratios (Wheeless *et al.*, 1984), measurement of DNA content (Tsou *et al.*, 1984; Fujii *et al.*, 1984; Barrett *et al.*, 1979; Tucker, 1979; Sprenger & Witte, 1979), DNA content plus total protein (Linden *et al.*, 1979) and image analysis assaying DNA and chromatin 'texture' (Al & Ploem, 1979; Smeulders *et al.*, 1979). These methods are based on either morphology or non-specific biochemical markers (DNA and total protein). This may not always be sufficient to make a reliable distinction as morphology, DNA content and chromatin 'texture' need not reflect the malignant phenotype. Our methods are being directed towards biochemical assays in normal and malignant cells which may reflect either qualitative or quantitative differences in specific proteins which are thought to play a part in growth regulation and proliferation control. The c-*myc* gene product is one such protein (Hann *et al.*, 1985; Kelly *et al.*, 1983, 1984; Makino *et al.*, 1984; Rabbitts *et al.*, 1985) and combinations of this technique with some of those mentioned above will undoubtedly result in more accurate automated screening procedures.

We thank Dr John Goepel, Weston Park Hospital, Sheffield for providing the biopsy specimens.

### References

AL, I. & PLOEM, J.S. (1979). Detection of suspicious cells and rejection of artefacts in cervical cytology using the Leyden Television Analysis system. *J. Histochem. Cytochem.*, **27**, 629.

BARRETT, D.L., JENSEN, R.H., KING, E.B., DEAN, P.N. & MAYALL, B.H. (1979). Flow cytometry of human gynaecologic specimens using log chromomycin A3 fluorescence and log 90 degrees light scatter. *J. Histochem. Cytochem.*, **27**, 573.

DOOLITTLE, R.F., HUNKERPILLER, M.W., HOOD, L.E. & 4 others. (1983). Simian sarcoma virus *onc* gene, v-*sis*, is derived from the gene (or genes) encoding a platelet derived growth factor. *Science*, **211**, 275.

DOWNWARD, J., YARDEM, Y., MAYES, E. & 6 others. (1984). Close similarities of epidermal growth factor receptor and v-*erb* B oncogene protein sequences. *Nature*, **307**, 521.

EVAN, G. & HANCOCK, D. (1985). Nuclear structures containing $p62^{c\text{-}myc}$. *Cell*, **43**, 253.

EVAN, G., LEWIS, G.K., RAMSAY, G. & BISHOP, J.M. (1985). Isolation of monoclonal antibodies specific for human and mouse proto-oncogene products. *Mol. Cell Biol.*, **5**, 3610.

ELIAS-JONES, J., HENDY-IBBS, P., COX, H., EVAN, G.I. & WATSON, J.V. (1986). Cervical brush biopsy specimens suitable for DNA and oncoprotein analysis using flow cytometry. *J. Clin. Pathol.*, **39**, 577.

FORGACS, I.C., SUNDARESAN, V., WIGHT, D.G.D. & 4 others. (1987). Abnormal c-*myc* oncoportein expression in dysplasia and carcinoma associated with ulcerative colitis. *Gut* (In press).

FUJII, T., CRUM, C.P., WINKLER, B., FU, Y.S. & RICHART, R.M. (1984). Human papillomavirus infection and cervical intraepithelial neoplasia: histopathology and DNA content. *Obstet. Gynaecol.*, **63**, 99.

GREEN, S., WALTER, P., KURMAN, V. & 4 others. (1986). Human oestrogen receptor cDNA: sequence, expression and homology to v-*erb*-A. *Nature*, **320**, 134.

GREENBERG, M.E. & ZIFF, E.B. (1984). Stimulation of 3T3 cells induces transcription of the c-*fos* proto-oncogene. *Nature*, **311**, 433.

HANN, S.R., THOMPSON, C.B. & EISENMAN, R.N. (1985). c-*myc* oncogene protein is independent of the cell cycle in human and avian cells. *Nature*, **314**, 366.

HEADLEY, D.W., FRIEDLANDER, M.I., TAYLOR, I.W., RUGG, C.A. & MUSGROVE, E.A. (1983). Method for analysis of cellular DNA content of paraffin-embedded pathological material using flow cytometry. *J. Histochem. Cytochem.*, **31**, 1333.

KELLY, K., COCHRAN, B.H., STILES, C.D. & LEDER, P. (1983). Cell specific regulation of the c-*myc* gene by lymphocyte mitogens and platelet derived growth factor. *Cell*, **35**, 603.

KELLY, K., COCHRAN, B.H., STILES, C.D. & LEDER, P. (1984). The regulation of c-*myc* by growth signals. *Curr. Topics Microbiol. Immunol.*, **113**, 117.

LINDEN, W.A., OCHLICH, K., BAISCH, H. & 7 others. (1979). Flow cytometric prescreening of cervical smears. *J. Histochem. Cytochem.*, **27**, 529.

MAKINO, R., HAYASHI, K.A. & SUGIMURA, T. (1984). c-*myc* is induced in rat liver at a very early stage of regeneration or by cycloheximide treatment. *Nature*, **310**, 697.

NIMAN, H.L., HOUGHTEN, R.A., WALKER, L.E. & 4 others. (1983). Generation of protein-reactive antibodies by short peptides in an event of high frequency: Implications for the structural basis of immune recognition. *Proc. Natl. Acad. Sci.*, **80**, 4949.

RABBITTS, P.H., WATSON, J.V., LAMOND, A. & 7 others. (1985). Metabolism of c-*myc* gene products: c-*myc* mRNA and protein expression in the cell cycle. *Embo. J.*, **4**, 2009.

RIOU, G., BARROIS, M., TORDJMAN, I., DUTRONQUAY, V. & ORTH, G. (1984). Presence de genomes de papillonavirus et amplification des oncogenes c-*myc* et c-Ha-*ras* dans des cancers envahissants du col de l'uterus. *C. R. Acad. Sc. Paris*, **299**, 575.

SCHERR, C.J., RETTENMIER, C.W., SACCA, R., ROUSSEL, M.F., LOOK, A.T. & STANLEY, E.R. (1985). The c-*fms* proto-oncogene product is related to the receptor for the mononuclear phagocyte growth factor, CSF 1. *Cell*, **41**, 665.

SIKORA, K., CHAN, S., EVAN, G. & 4 others. (1987). c-*myc* oncogene expression in colorectal cancer. *Cancer*, (In press).

SIKORA, K., EVAN, G., STEWART, J. & WATSON, J.V. (1985). Detection of c-*myc* oncoprotein in testicular cancer. *Br. J. Cancer*, **52**, 171.

SMEULDERS, A.W., LEYTE-VELDSTRA, L., PLOEM, J.S. & CORNELISSE, C.J. (1979). Texture analysis of cervical cell nuclei by segmentation of chromatin patterns. *J. Histochem. Cytochem.*, **27**, 199.

SPRENGER, E. & WITTE, S. (1979). The diagnostic significance of nuclear deoxyribonucleic acid measurement in automated cytology. *J. Histochem. Cytochem.*, **27**, 520.

STEWART, J., EVAN, G.I., WATSON, J.V. & SIKORA, K.E. (1986). Detection of the c-*myc* oncogene product in colonic polyps and carcinomas. *Br. J. Cancer*, **53**, 1.

TSOU, K.C., HONG, D.H., VARELLO, M., GIUNTOLL, R., WHEELER, J.E., ATKINSON, B.F., MANGAN, C. & MIKUTA, J. (1984). Flow cytometric DNA analysis as a diagnostic aid for cervical condyloma and cancer. *Cancer*, **54**, 1778.

TUCKER, J.H. (1979). An image analysis system for cervical cytology automation using nuclear DNA content. *J. Histochem. Cytochem.*, **27**, 613.

WAKELAM, M.J.O., DAVIES, S.A., HOUSLAY, M.D., McKAY, I., MARSHALL, C.J. & HALL, A. (1986). Normal $p21^{N\text{-}ras}$ couples bombesin and other growth factor receptors to inositol phosphate production. *Nature*, **323**, 173.

WATERFIELD, M.D., SCRACE, G.T., WHITTLE, N., STROOBANT, P., JOHNSON, A., WASTESON, A., WESTERMARK, B., HUANG, J. & DEUEL, T.F. (1983). Platelet derived growth factor is structurally related to the putative transforming protein $p28^{sis}$ of simian sarcoma virus. *Nature*, **304**, 35.

WATSON, J.V. (1980). Enzyme kinetic studies in cell populations using fluorogenic substrates and flow cytometric techniques. *Cytometry*, **1**, 143.

WATSON, J.V. (1981). Dual laser beam focussing for flow cytometry through a single crossed cylindrical lens pair. *Cytometry*, **2**, 14.

WATSON, J.V. (1985). A method for improving light collection by 600% from square cross section flow cytometry chambers. *Br. J. Cancer*, **51**, 433.

WATSON, J.V., STEWART, J., EVAN, G., RITSON, A. & SIKORA, K. (1986). The clinical significance of flow cytometric c-*myc* oncoprotein quantitation in testicular cancer. *Br. J. Cancer*, **53**, 331.

WATSON, J.V., SIKORA, K.E. & EVAN, G.I. (1985). A simultaneous flow cytometric assay for c-*myc* oncoprotein and cellular DNA in nuclei from paraffin embedded material. *J. Immunol. Meths.*, **83**, 179.

WHEELESS, L.L., PATTEN, S.F., BERKAN, T.K. & 5 others. (1984). Multidimensional slit-scan prescreening system: preliminary results of a single blind clinical study. *Cytometry*, **5**, 1.